# A Potent Combination Microbicide that Targets SHIV-RT, HSV-2 and HPV

**DOI:** 10.1371/journal.pone.0094547

**Published:** 2014-04-16

**Authors:** Larisa Kizima, Aixa Rodríguez, Jessica Kenney, Nina Derby, Olga Mizenina, Radhika Menon, Samantha Seidor, Shimin Zhang, Keith Levendosky, Ninochka Jean-Pierre, Pavel Pugach, Guillermo Villegas, Brian E. Ford, Agegnehu Gettie, James Blanchard, Michael Piatak, Jeffrey D. Lifson, Gabriela Paglini, Natalia Teleshova, Thomas M. Zydowsky, Melissa Robbiani, José A. Fernández-Romero

**Affiliations:** 1 Center for Biomedical Research, Population Council, New York, New York, United States of America; 2 Aaron Diamond AIDS Research Center, Rockefeller University, New York, New York, United States of America; 3 Tulane National Primate Research Center, Tulane University, Covington, Louisiana, United States of America; 4 AIDS and Cancer Virus Program, Leidos Biomedical Research, Inc. (Formerly SAIC-Frederick, Inc.), Frederick National Laboratory, Frederick, Maryland, United States of America; 5 Instituto de Virología J.M.Vanella-Facultad de Ciencias Médicas-Universidad Nacional de Córdoba, Córdoba, Argentina; University of California, San Francisco, United States of America

## Abstract

Prevalent infection with human herpes simplex 2 (HSV-2) or human papillomavirus (HPV) is associated with increased human immunodeficiency virus (HIV) acquisition. Microbicides that target HIV as well as these sexually transmitted infections (STIs) may more effectively limit HIV incidence. Previously, we showed that a microbicide gel (MZC) containing **M**IV-150, zinc acetate (**Z**A) and carrageenan (**C**G) protected macaques against simian-human immunodeficiency virus (SHIV-RT) infection and that a ZC gel protected mice against HSV-2 infection. Here we evaluated a modified MZC gel (containing different buffers, co-solvents, and preservatives suitable for clinical testing) against both vaginal and rectal challenge of animals with SHIV-RT, HSV-2 or HPV. MZC was stable and safe *in vitro* (cell viability and monolayer integrity) and *in vivo* (histology). MZC protected macaques against vaginal (p<0.0001) SHIV-RT infection when applied up to 8 hours (h) prior to challenge. When used close to the time of challenge, MZC prevented rectal SHIV-RT infection of macaques similar to the CG control. MZC significantly reduced vaginal (p<0.0001) and anorectal (p = 0.0187) infection of mice when 10^6^ pfu HSV-2 were applied immediately after vaginal challenge and also when 5×10^3^ pfu were applied between 8 h before and 4 h after vaginal challenge (p<0.0248). Protection of mice against 8×10^6^ HPV16 pseudovirus particles (HPV16 PsV) was significant for MZC applied up to 24 h before and 2 h after vaginal challenge (p<0.0001) and also if applied 2 h before or after anorectal challenge (p<0.0006). MZC provides a durable window of protection against vaginal infection with these three viruses and, against HSV-2 and HPV making it an excellent candidate microbicide for clinical use.

## Introduction

HIV, HPV and HSV-2 constitute the three major viral STIs, and infection with HPV [1] or HSV-2 [2] increases HIV susceptibility. Like HIV, these viruses have no cure. Although quadrivalent (targeting genotypes 6, 11, 16 and 18) and divalent (targeting genotypes 16 and 18) preventive vaccines against HPV are commercially available, HPV vaccination rates are currently low due to many challenges, including parental autonomy and cost [3]. Moreover, other important HPV types are not targeted, and the vaccine requires a cold supply chain, which limits its use in developing countries [3] and confirms the need for other preventive modalities, such as microbicides with anti-HPV activity.

Concrete evidence now substantiates the ability of a microbicide to inhibit both HIV and HSV-2. The Phase 2b CAPRISA 004 trial demonstrated reduced vaginal acquisition of both viruses in the presence of 1% tenofovir (TFV) gel and set the stage for advancing microbicide development against HIV and broadening the target to include co-pathogens [4].

The most advanced products in the microbicide pipeline are based on single active pharmaceutical ingredients (APIs), specifically reverse transcriptase inhibitors (RTIs) [4], [5], [6] (http://www.mtnstopshiv.org/node/4546). Although single API products are still being advanced, the transition to products composed of multiple drugs with differing modes of action is gaining momentum; especially as novel formulations, vehicles and delivery systems are developed to enable the release of diverse compounds [7], [8]. Double and triple combinations of APIs may enhance both the potency and breadth of anti-HIV protection as they show synergistic activity [9] and cover many drug resistant isolates [10]. Multipurpose prevention technologies (MPTs) that simultaneously target multiple sexual and reproductive health needs will improve health and save resources (http://cami-health.org/documents/2012-SAWG-Report-FinalReport.pdf). MPTs that combine APIs to prevent different STIs and/or unwanted pregnancy are being developed in different delivery systems, predominantly intravaginal rings (IVRs) (http://www.cami-health.org/documents/Microbicides-and-Devices.pdf). Although IVRs offer women a discrete sustained-release microbicide alternative, giving women options is of paramount importance for adherence [11]. On-demand products, like gels, are still a priority (http://cami-health.org/documents/2012-SAWG-Report-FinalReport.pdf) and have the potential to be used both vaginally and rectally.

In initial testing, the first generation MZC gel (containing the non-nucleoside reverse transcriptase inhibitor (NNRTI) **M**IV-150, **Z**inc acetate and **C**arrageenan) provided macaques up to 8 h of complete protection against vaginal infection with SHIV-RT [12], [13]. ZC gels were highly effective at preventing high dose HSV-2 vaginal and anorectal infection in mice [14] and even significantly reduced vaginal SHIV-RT infection (though less effectively than MZC [12]). Additional data suggest that CG has activity against HPV [15], [16], [17], [18]. Herein we demonstrate that a new formulation of MZC modified for safety in humans is indeed safe, and blocks vaginal SHIV-RT infection in macaques as well as vaginal and rectal HSV-2 and HPV infection in mice. Thus, MZC is a unique formulation protecting against HIV, HSV-2, and HPV, warranting advancement to clinical testing.

## Materials and Methods

### Ethics statement for animal procedures

Housing and care of adult female Indian rhesus macaques (*Macaca mulatta*) complied with the regulations under the Animal Welfare Act, and the Guide for the Care and Use of Laboratory Animals [19] at Tulane National Primate Research Center (TNPRC; Covington, LA). All macaque studies were approved by the Institutional Animal Care and Use Committee (IACUC) of the TNPRC for macaques (#A4499-01) and complied with animal care procedures [19], [20], receiving full accreditation by the Association for Accreditation of Laboratory Animal Care (AAALAC #000594). All mouse studies were approved by the IACUC of the Comparative Bioscience Center (CBC) at The Rockefeller University (IACUC protocol # 10019, 10039 and 12563).

The macaques were socially housed until challenge and then separated until infection status was determined. Macaques with similar infection status (either infected or uninfected) were then socially housed again. The housing restrictions were scientifically justified and approved by the IACUC as part of the protocol review. All the animals on this study were fed commercially prepared monkey chow twice daily and supplemental foods were provided in the form of fruit, vegetables, and foraging treats as part of the TNPRC environmental enrichment program. The TNPRC environmental enrichment program is reviewed and approved by the IACUC semiannually. Extensive efforts are made to find compatible pairs for every study group, and additional environmental enrichment is provided through a variety of food supplements and physical complexity of the housing space. A team of 11 behavioral scientists monitored the well-being of the animals and provided direct support to minimize stress during the study period. The macaques were anesthetized with tiletamine/zolazepam (8 mg/kg body weight) prior to blood draws and biopsies and treated with buprenorophine (0.01 mg/kg body weight) for analgesia. Preemptive and post-procedural anesthetics and analgesics are required by the TNPRC division of veterinary medicine for procedures that would likely cause more than momentary pain or distress in humans undergoing the same procedure. No macaques were sacrificed in the studies.

All mouse care procedures were in compliance with the regulations detailed under the Animal Welfare Act [20] and the Guide for the Care and Use of laboratory Animals [19]. Veterinarians at the TNPRC Division of Veterinary Medicine and at the CBC at The Rockefeller University monitored animals regularly to minimize any distress or pain.

### Formulations

MZC: Step 1: A 4 L Ross double planetary mixer was charged with 2678 ml of sterile purified water and 3.93 g of sodium acetate. The solution was heated for 5 min at 69°C with stirring at 40 rpm. CG (93 g) was added and the mixture was stirred for 3 h at 69°C. Step 2: The formulation was cooled to 25°C. Step 3: A solution of 9 g of ZA in sterile purified water was added to the formulation from step 2 and stirred for 20 min at 40 rpm. A solution of 6 g of methyl paraben and 55.6 mg of MIV-150 in propylene glycol (60 g) was then added and the solution was stirred for an additional 1 h at 40 rpm. MIV-150 (C_19_H_17_FN_2_O_3_) is a potent NNRTI that belongs to the group of phenethylthiazolylthiourea derivatives [21]. Step 4: If necessary, the pH of the formulation was adjusted to 6.8 to 7.0 with either 1N NaOH or 1N HCl. Step 5: The mixture was stirred for 15 min under vacuum to remove air bubbles. The following lot numbers were used in our studies: 110523A1005ML, 120120A1005MR, 110921A1005ML, 120926A1005ML, 110606A1005MR and 110523A1005ML. A control 3% (w/v) CG vehicle prepared under the same conditions but in the absence of ZA and MIV-150 was used in several experiments (Lot numbers 120111A525MR, 110509A525MR, and 110512A525). Hydroxyethylcellulose (HEC) gel was formulated at Clean Chemical Sweden (Borlänge, Sweden) as described by Tien et al [22].

### Stability studies

Twenty-five gram aliquots of test gel were placed in 30 ml polypropylene bottles that were stored under the following conditions: 30°C/65% relative humidity, 40°C/75% relative humidity, and 50°C/ambient humidity. Bottles were removed at scheduled times and the gel analyzed for methyl paraben content, MIV-150 content, osmolality, pH, viscosity, and Zn^2+^ content as previously described [14].

### *In vivo* gel distribution in macaques

To assess the distribution and spread of MZC in the macaque reproductive tract, MRI was employed. All MRI examinations (3D SPGR T1 weighted gradient echo with fat saturation) were performed at 2 and 24 h after 2 ml MZC was instilled into the vagina. Animals were anesthetized with tiletamine/zolazepam (8 mg/kg IM) for the MRI scan (GE Signa Horizon LX1.5T with Software 9.1; GE Healthcare, Port Washington, NY). Sagittal and transverse images were taken to assess spread of the gel throughout the reproductive tract.

### Cells and Viruses

HeLa and Caco-2 cells (ATCC, Rockville, MD) were cultured and/or differentiated as previously described [23]. Human peripheral blood mononuclear cells (PBMCs) were isolated from healthy blood donors (New York Blood Center, Long Island City, NY) and the 3×3 stimulation was performed as previously described by Trkola et al [24]. After activation, PBMCs were grown in fresh stimulation media consisting of RPMI 1640 (Life Technologies), 10% FBS, antibiotics at a final concentration of 50 U/ml of penicillin, 50 µg/ml streptomycin and recombinant IL-2 at 20 U/ml (Roche, Indianapolis, IN).

*L. jensenii*, *L. crispatus* and *C. albicans* strain SC5413 (ATCC) were propagated as previously described [23].

#### HIV and SHIV-RT

Table S1 summarized the HIV-1 laboratory strains, primary isolates, MDR isolates/clones and SHIV-RT virus used in our experiments. The original SHIV-RT stocks were grown in PHA activated human PBMCs. SHIV-RT stocks were re-titered using 174×CEM cell line (NIH AIDS Research & Reference Reagent Program), while HIV stocks were re-titered using 3×3 activated human PBMCs. TCID_50_ were calculated using the Reed and Muench formula. Aliquots of virus stocks were stored at −80°C.

#### HSV-2 G strain

The virus was propagated in Vero cells (ATCC) and titered using the plaque formation assay on Vero cells as previously described [25]. Aliquots of virus stock were stored at −80°C.

#### HPV PsVs

HPV-16, 18 and 45 PsVs were produced following the NCI protocol published on Dr. John Schiller's laboratory website http://home.ccr.cancer.gov/Lco/. For this purpose, 293TT cells were obtained from NCI at Frederick, MD, and the following plasmids were generously provided by Dr. John Schiller or purchased through Addgene, Cambridge, MA: p16shell and Addgene plasmids 37321 [17], 37323 [17] and 37328 [26]. Aliquots of virus stocks were stored at −80°C.

The pCLucf/HPV PsV stocks were titered by quantitative PCR (qPCR) to assess the reporter plasmid (pCLucf # 37328) copy number. Primers that target the EGFP region of pCLucf were designed using ABI's Primer Express software. Each reaction consisted of 12.5 µl of 2× ABsolute Blue qPCR Sybr Green (Thermo Scientific, Rockford, IL) master mix, 70 nM each EGFP primer (forward GAGCTGAAGGGCATC GACTT and reverse CTTGTGCCCCAGGATGTTG) and 5 µl of a 1∶1000 dilution of PsV stock in a reaction volume of 25 µl. Cycling conditions consisted of an initial denaturation step at 95°C for 15 min followed by 40 cycles of alternating 95°C for 15 seconds, 60°C for 30 seconds and 72°C for 30 seconds. Fluorescence was detected during the elongation stage. A melting curve step was added after the end of the cycling to assess specificity of the reaction. For standard curves, qPCR was performed on a 10-fold dilution series of purified pCLucf plasmid ranging from 10^8^ copies to 10 copies per reaction. Reactions were performed using an ABI ViiA 7 thermal cycler.

### *In vitro* toxicity and anti-HPV PsV activity

HeLa cells were plated at 10^4^ cells/well in 100 µl of medium and incubated overnight at 37°C, 5% CO_2_ and 98% humidity. MZC or CG was diluted in medium to obtain 2× dilutions of the appropriate dilution range to test (0.000067 to 0.00000001). The cell culture media was then removed from the microplates and replaced with 50 µl of the dilutions of each formulation or 50–100 µl of medium for virus and cell controls. Dilutions were tested in triplicate. HPV PsV stocks were thawed on ice and diluted to 10^7^ copies/ml. Fifty μl of PsV (5×10^5^ copies) were added to all wells with the exception of cell controls and incubated for 72 h at 37°C, 5% CO_2_ and 98% humidity. The cells were lysed to detect luciferase activity using the Pierce Firefly Luciferase Glow Assay with Pierce Firefly Signal Enhancer (Thermo Scientific) as described by the manufacturer. Luminescence was read on a Gemini EM microplate reader (emission 542 nm) using Softmax Pro 3.2.1 software. Cytotoxicity for each gel formulation was estimated using the XTT assay [27], [28]. For this purpose, the antiviral assay was mimicked but in the absence of virus. CC_50_ and IC_50_ values were calculated using a dose-response-inhibition analysis on GraphPad Prism v5.0c software. Therapeutic indexes (TI = CC_50_/IC_50_) were calculated.

### *In vitro* toxicity and anti-HIV activity in PBMCs

Activated PBMCs (2×10^6^/ml) were treated for 1 h with dilutions of gels (triplicates) before adding 100 TCID_50_ of virus and incubating overnight. To compare the anti-HIV activity of MZC and CG *in vitro* this had to be presented based on gel dilution, since there is no MIV-150 (which is the active component in this assay) in the CG control. The supernatant was replaced with fresh stimulation media on d 1 and 4 post infection. The p24 level in the supernatant was tested on d 7 after infection by p24 ELISA (Zeptometrix, Buffalo, NY). Cytotoxicity for each gel formulation was estimated using the XTT assay by mimicking the antiviral assay in the absence of virus [27], [28]. CC_50_ and IC_50_ values were calculated using a dose-response-inhibition analysis on GraphPad Prism v5.0c software. Therapeutic indexes (TI = CC_50_/IC_50_) were estimated.

### *In vitro* toxicity against lactobacilli and *C. albicans*

Diluted gel formulations and Penicillin/Streptomycin (P/S; P = 100 U/ml and S = 100 µg/ml; Life Technologies) or amphotericin B (Amph.B; 50 µg/ml; Life Technologies) were tested for toxicity against lactobacilli and *C. albicans* as previously described [23]. The lactobacilli and *C.albicans* were considered sensitive to killing if the formulations caused a viability reduction equal to or greater than 1 log_10_ [29]_._

### Transepithelial electrical resistance (TEER)

The TEER assay was performed as previously described [27], except that the media was replaced on the day of the assay with fresh DMEM without phenol red (Life Technologies) supplemented with MITO + Serum Extender (BD Biosciences). All formulations were diluted 1∶10 in the above media and 300 µl were applied in triplicate in the upper chambers. Resistance readings were performed at 0, 2, 4 and 6 h.

### Animal studies to measure safety

#### Histology

Medroxypregesterone acetate (Depo-Provera, depo)-treated Balb/C mice or fasted and anesthetized (Ketamine/Xylazine solution) mice were given formulations vaginally or rectally, respectively. Dulbecco's Phosphate-Buffered Saline (D-PBS) was used as a no damage reference and Gynol II (www.drugstore.com) as a reference for tissue damage. The mice were sacrificed at 1, 6, and 24 h after gel installation, and vaginal or rectal tissue was collected and processed [14]. Morphological analysis was performed using hematoxylin and eosin (H&E) staining followed by examination of the stained tissue sections to evaluate the mucosal architecture.

#### HSV-2 increased susceptibility model

Following our established method [14], 10 µl of each formulation was applied intravaginally to depo-treated Balb/C mice daily for 7 d, and 12 h after the last application, mice were challenged with 10 µl of 2 × 10^3^ pfu of HSV-2 G. In each experiment, a placebo group (D-PBS) was used in addition to CG to compare to MZC. Beginning on d 4 after inoculation, mice were examined and scored daily for 21 d total. Animals with symptoms of infection (hind limb paralysis, erythema, hair loss, and/or swelling in the vaginal area) were scored as infected and euthanized.

### Animal studies to measure efficacy

#### Macaque efficacy and PK studies

We performed the studies in adult female Indian rhesus macaques (*Macaca mulatta*) ranging from 4–12 years old and weighing from 4–10 kg. Animals tested negative for simian type D retroviruses, simian T cell leukemia virus-1, and SIV prior to use in the efficacy studies. Animals were anesthetized with ketamine-HCL (10 mg/kg) before EDTA blood samples were taken (no more than 10 ml/kg/month) at the indicated time points pre and post-virus challenge. Preemptive and post-procedural analgesia was required for procedures that would likely cause more than momentary pain or distress in humans undergoing the same procedures. 5 wks before vaginal virus challenge, animals received a single 30 mg i.m. injection of depo.

For the group challenged vaginally, depo-treated macaques were atraumatically dosed vaginally with 2 ml of MZC or CG using a pliable French catheter for 14 consecutive days. 24 or 8 h after the last application, they were challenged vaginally with 0.5 ml of 10^3^ TCID_50_ SHIV-RT (SIVmac239 and HIV-1_HxB2_ RT). All animals were maintained in a supine position for 20–30 min post-challenge to allow absorption of virus. Not only does this approach evaluate the activity of MZC (vs. CG), since ∼65% of the CG-treated animals become infected it also provides a measure of safety (i.e., that there is not >65% infection in the MZC group, especially after repeated dosing). In the group challenged rectally, macaques were dosed rectally with 3 ml of MZC or CG using a pliable French catheter 1 h before rectal challenge with 0.5 ml of 10^3^ TCID_50_ SHIV-RT. Monkeys were anesthetized with ketamine-HCl (10 mg/kg) prior to these procedures. Individual animal information is summarized in Table S2. Blood samples were transported overnight from the TNPRC to our laboratories at the Population Council for processing and analysis.

To approximate *in vivo* levels of MIV-150 in efficacy experiments (direct sampling not possible), vaginal or rectal biopsies, swabs and plasma samples were collected from a separate group of animals treated with gels as in the efficacy studies and then MIV-150 in those samples was quantified. Depo-treated animals received daily applications of MZC vaginally for 2 wks. Plasma was collected at different time points up to 24 h post-gel (n = 6), and vaginal and cervical tissues were collected 8 h (n = 12) and 24 h (n = 6) after the last gel application. Animals received a single application of MZC rectally, and plasma (n = 12) and rectal tissue (n = 6) were collected 1 h later. Vaginal swabs were collected 8 h (n = 6) and 24 h (n = 6) after last vaginal gel application, while rectal swabs were collected 1 h (n = 6) after single rectal gel application. Vaginal/rectal fluids were collected using a Merocel eye spear sponge inserted into the vaginal/rectal vault. The tip of the sponge was saturated in 1 ml sterile saline (without serum or antibiotics) or PBS with 1% FBS and antibiotics (pen/strep) prior to insertion to prevent injury of the mucosa. The sponge was left in the vagina/rectal cavity for about 5 min to absorb mucosal fluid and was rotated several times to absorb more mucosal fluid prior to removal. Upon removal, the saturated sponge was placed immediately back into the tube containing the 1 ml PBS or saline. The unknown volume of native cervicovaginal or rectal fluid was not accounted for in the final calculations of API concentrations.

We quantified MIV-150 in swabs by RIA [LLOQ, 1 ng/ml (2.7 nM)] [12], and in plasma by LCMS/MS [LLOQ, 20 pg/ml (54 pM)] as previously described [13]. We developed and validated an LCMS/MS procedure for MIV-150 quantification in tissue. Tissue samples were processed by mixing 120 µl of water, 280 µl of ACN and about 20 mg tissue sample in Lysing Matrix A tubes (MP Biomedicals, Solon, OH). Samples were homogenized at 60 speed for 40 seconds, 3 times using a FastPrep-24 homogenizer (MP Biomedicals, Solon, OH), followed by sonication for 20 min (Branson 3510, Danbury, CT). Samples were centrifuged for 5 min at 5,000 rpm, and then supernatant was transferred into a conical polypropylene tube (USA Scientific, Orlando, FL), centrifuged for 10 min at 13,000 rpm, and used for MIV-150 quantitation. MIV-160 (a related NNRTI with similar chemical properties provided by Drs. Bo Öberg and Disa Böttiger, Medivir) was used as the internal standard at 2 ng/ml. The compounds were separated using a ACQUITY UPLC BEH analytical column (1.7 µm, 2.1 × 50 mm) (Waters, Milford, MA) using gradient elution with a mobile phase consisting of 5% ACN in water [A] and ACN [B], with addition of 0.1% acetic acid to both [A] and [B], at a flow rate of 0.5 ml/min. The retention times for MIV-150 and MIV-160 were 0.8 and 0.57 min, respectively, with a total run time of 5 min. The analytes were detected with a Xevo-TQs (Waters, Milford, MA) triple quadrupole mass spectrometer in positive electrospray ionization mode using multiple reaction monitoring (MRM). The extracted ions monitored following MRM transitions were m/z 369.296→224.114 for MIV-150 and m/z 343.2→198.06 for MIV-160. The LLOQ was 111 fg on the column (0.25 pg/mg of tissue). The overall intraday and interday assay RSD% and RE% were <15%.

#### SIVgag PCR

Dry PBMC pellets (5×10^6^ cells) were frozen and stored at −80°C until needed [12]. Nested PCR was performed on lysed pellets to determine the presence of SIV*gag* DNA [30].

#### RT-PCR

Plasma viral RNA copy numbers were determined by quantitative RT-PCR [31]. Animals were defined as infected when they recorded >10^3^ RNA copies/ml in at least 2 consecutive samples within the 20 weeks post infection follow up period.

#### SIV Ab responses

SIV-specific Abs were monitored by ELISA [12], [32]. Ab positivity was defined as having positive OD values over the baseline at 4 or 8 weeks post-challenge.

#### Detection of SHIV-RT drug resistance mutations

Drug resistance mutations (DRMs) in the RT gene of SHIV-RT circulating in animals that became infected during the study were screened by sequencing plasma virus RNA using the previously described method [33].

#### High HSV-2 dose challenge in mice

Vaginal and anorectal high HSV-2 dose challenge was performed as previously described [14].

#### Window of protection with low HSV-2 dose in mice

Depo-treated mice were given 10 µl of test formulation intravaginally at 8 or 4 h prior to HSV-2 infection as well as 2, 4 or 8 h after HSV-2 infection. Ketamine/Xylazine treated mice were given 20 µl of test formulation in the anorectal area at the same time points described for the vaginal application. All the mice were challenged intravaginally with 10 µl of HSV-2 G (5×10^3^ pfu/mouse) or in the anorectal area with 10 µl of HSV-2 G (10^5^ pfu/mouse). In each experiment, a placebo group (D-PBS) was used in addition to the CG vehicle-only gel to compare to MZC. All mice were examined and scored as described in the “HSV-2 increased-susceptibility model” section.

#### HPV-16 PsV vaginal challenge in mice

The assay was performed to test anti-HPV activity of the MZC formulation following the procedure described by Roberts et al. [17], with the only difference that formulation was not pre-mixed with virus inoculum before vaginal application. Instead, 10 µl of test formulation was inserted intravaginally 24 h, 8 h or 10 min before challenging with 8×10^6^ copies/10 µl of HPV-16 PsV, as well as 0.5 or 2 h after PsV challenge. In all experiments, Conceptrol was applied 6 h before challenge as previously described for the HPV PsV vaginal model [17]. The Conceptrol treatment promotes abrasions in the epithelium, allowing the binding of HPV PsV to the basement membrane. *In vivo* luciferase expression was measured 24 h after intravaginal challenge by anesthetizing the animals with isoflurane (Aerrane, Deerfield, IL), applying 20 µl of D-luciferin (Caliper Life Sciences, Hopkinton, MA) intravaginally, incubating 3 min and performing *in vivo* imaging in the IVIS Lumina (Xenogen, Alameda, CA). Luminescence signal was expressed as mean value in radiance.

#### HPV-16 PsV anorectal challenge in mice

Six to eight-week-old female Balb/C mice (Charles River Laboratories) were fasted for 24 h prior to the anorectal HPV-16 PsV challenge, but food and water were available ad libitum following initial HPV-16 PsV challenge. Six hours before PsV challenge, the animals were anesthetized to both immobilize them and prevent defecation using an IP injection of 100 µl of a solution containing: 3 ml of dissolved Ketamine (Fort Dodge Laboratories, Fort Dodge IA), 320 µl of Xylazine (Miles Inc., Shawnee Mission KS) and 12.48 ml D-PBS. The next step was to immediately apply 30 µl of Conceptrol gel (Revive Personal, Madison, NJ) in the anorectal area to promote abrasions in the epithelium [17]. Mice were anesthetized with isoflurane 6, 4 or 1 h later and challenged with 8×10^6^ copies/10 µl D-PBS of HPV-16 PsV to determine the optimal time for Conceptrol treatment. One hour after Conceptrol treatment rendered the best luminescence signal and was chosen to test the MZC formulation vs. HEC placebo. Twenty μl of gel formulations were applied at different time points before (0.5, 2 and 8 h) or after (0.5 and 2 h) PsV challenge to test anti-HPV PsV activity. *In vivo* luciferase expression was measured as described in the vaginal model but anesthetizing the animals with ketamine/xylazine 24 and 48 h after PsV challenge and applying 20 µl of D-luciferin in the anorectal area. All the anorectal inoculations were performed by introducing a tip loaded with the sample in the rectum about 0.5 cm and dispensing while retracting the tip towards the anal canal.

#### CG PK in mice and CG detection

PK was performed under the same conditions as the efficacy studies for vaginal and rectal HPV PsV challenge but in the absence of HPV PsV. For this purpose, vaginal and rectal washes were collected for detection of CG from groups of six or eight animals by washing with D-PBS (200 µl) at 0.5, 1, 2, 4, 8 or 24 h after applying 10 µl (vaginal) or 20 µl (rectal) of MZC. Vaginal or rectal washes from control mice that did not receive MZC (n = 6 or 8 per time point) were also collected. The unknown volume of native cervicovaginal or rectal fluid was not accounted for in the final calculations. The in house CG ELISA was performed to detect CG [LLOQ =  40 ng/ml]. Ninety six-well maxisorp microtiter plates (Nunc, Roskilde, Denmark) were coated with 100 µl/well (2.5 µg/ml in D-PBS) of custom prepared rabbit anti-CG polyclonal IgG antibody (Pacific Immunology, Ramona, CA) overnight at 37°C. The plates were inverted to discard the coating antibody and immediately blocked with 200 µl/well of assay diluent (Zeptometrix) for 1 h at 37°C. The plates were then washed six times (300 µl/well) with washing buffer (Zeptometrix). A standard curve of CG in a range of 10,000 to 40 ng/ml was prepared in diluent assay using the 3% (w/v) CG gel formulation described in the formulation section. Vaginal and rectal wash samples were diluted 1∶1,000 and 1∶10,000 in assay diluent. All standards and samples were tested in duplicates 100 µl/well. The plates were incubated for 1 h at 37°C followed by a washing step as described above. One hundred μl/well (1 µg/ml) of custom prepared biotinylated rabbit anti-CG polyclonal IgG antibody (Pacific Immunology) were added to all wells but the blank, before incubating 1 h at 37°C. The plates were washed again and HRP-based detection system with TMB substrate (Zeptometrix) was used to complete the ELISA after applying the stop solution (Zeptometrix). Absorbance was measured at 405 nm with an Emax Molecular Devices microplate reader.

### Statistical analyses

Fisher's exact test was used for comparison of SHIV-RT infection frequency in the different treatment groups. Since the number of placebo macaques are limiting in our statistics, relevant historical controls (challenged identically and using the same virus stock) are included in addition to the real time controls as done previously [12], [13], [23]. Fisher's exact test was also used for comparison of mouse infection after challenge with HSV-2. The Mann Whitney U test was used for comparison of *in vivo* luciferase expression in the HPV PsV mouse model. Statistical analyses were performed using GraphPad Prism version 5.02 for Windows (GraphPad Software, San Diego, CA). P values <0.05 were taken as statistically significant.

## Results

### MZC is stable and safe

A side-by-side comparison of different properties of the first generation prototype gel used in previous studies [12], [13] and the modified gel evaluated in this study is shown in Table 1. Modified MZC was stable for 12 months at 30°C/65% RH (relative humidity) and 9 months at 40°C/75% RH. Even after 1 month at 50°C, MZC remained stable (Table 1). Previous studies have shown that CG-based gels are shear thinning spreading gels [14]. Magnetic resonance imaging (MRI) performed 2 h after vaginal MZC administration in macaques showed distribution of the gel throughout the vaginal vault; gel was no longer detected by 24 h post-gel application (Fig. S1). Additionally, no gel was detected in the endocervix or endometrium at any time point.

**Table 1:** Formulation attributes after long term stability testing.

Property	Prototype MZC Gel	Modified MZC Gel
CG	3.0%	3.1%
Lambda:kappa CG	94∶6	60∶40
Buffer	none	10 mM sodium acetate
Co-solvent	1% DMSO	2% Propylene glycol
MIV-150	50 µM	50 µM
ZA	14 mM	14 mM
Methyl paraben	0.2%	0.2%
pH	6.64	6.90
Osmolality	474 mOsmol/kg	447 mOsmol/kg
Viscosity (freshly prepared)	29,300 cP	29,900 cP
Viscosity (3 months at 30°C/65% RH)	NT	35,100 cP
Viscosity (6 months at 30°C/65% RH)	NT	36,200 cP
Viscosity (9 months at 30°C/65% RH)	NT	34,200 cP
Viscosity (12 months at 30°C/65%RH)	NT	32,600 cP
Viscosity (3 months at 40°C/75% RH)	NT	22,700 cP
Viscosity (6 months at 40°C/75% RH)	NT	28,200 cP
Viscosity (9 months at 40°C/75% RH)	NT	24,600 cP
Viscosity (1 month at 50°C/ambient)	NT	34,000 cP

Formulations were placed in 30 ml polypropylene bottles and stored at 30°C/65% RH, 40°C/75% RH, and 50°C/ambient humidity. The gels were analyzed at indicated times for pH, viscosity, osmolality, methyl paraben content, and ZA content. The specified acceptable ranges for each parameter are indicated in each column heading. NT  =  not tested.

Previous macaque studies showed that prototype MZC was safe, as determined by examining vaginal pH and the cytokines and chemokines secreted locally after repeated gel application [12]. In this study, we expanded the safety evaluation to include several *in vitro* and *in vivo* assays that allowed us to estimate potential damage to epithelial integrity and toxicity towards normal microflora.

**Figure 1:**
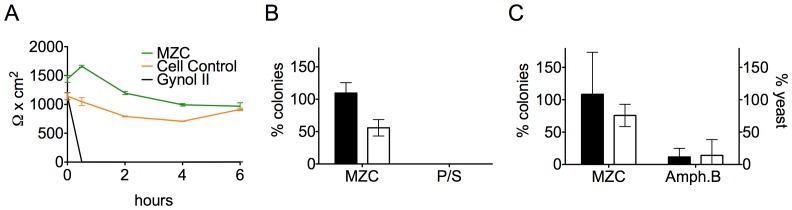
Modified MZC is safe *in vitro*. A) Caco-2 cell monolayer integrity. TEER was measured in differentiated Caco-2 cell monolayers after treatment with 1∶10 diluted formulations for 0-6 h. Means ± SD are shown from two independent experiments performed in triplicate. B) Lactobacilli *in vitro* viability. MZC toxicity was measured by treating *L. jensenii* (empty bars) and *L. crispatus* (filled bars) with 1∶10 diluted MZC vs. P/S for 30 min. The number of colonies (mean ± SD from three independent experiments) relative to saline 7.5% FBS, is shown as a percent. C) *C. albicans in vitro* viability. MZC toxicity was measured by incubating *C. albicans* yeasts with 1∶10 diluted MZC vs. Amph.B for 2 h. Left axis (filled bars) represents the numbers of colonies counted for each condition and expressed as % colonies relative to the Sabouraud dextrose broth (SB) control (set as 100%). Right axis (empty bars) is the number of viable yeasts counted and shown as the percentage relative to the SB-treated controls. Means ± SD of 5 independent experiments are summarized.

*In vitro*, MZC did not affect cell monolayer integrity as measured by TEER in differentiated Caco-2 monolayers for up to 6 h, in contrast with the rapid decrease of TEER values exerted by Gynol II (Fig. 1A). *Lactobacillus jensenii, lactobacillus crispatus* and *Candida albicans* viability was not significantly affected by MZC, whereas P/S and Amph.B both significantly decreased viability of the respective organisms (> 1 log_10_ reduction) (Fig. 1B and 1C). MZC also did not drive the differentiation of unicellular yeast form into the pathogenic multicellular filamentous form (Fig. 1C).

**Figure 2:**
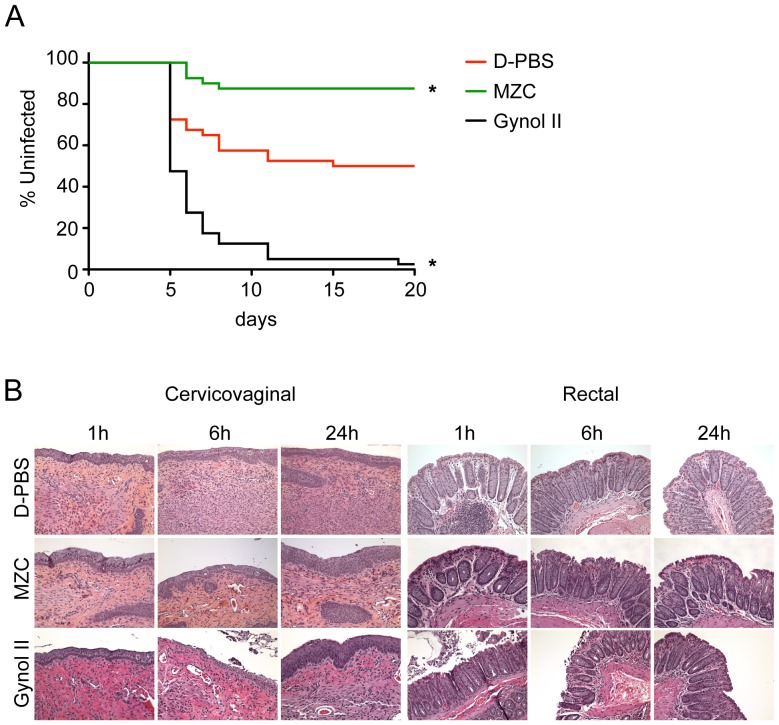
*In vivo* application of MZC does not compromise epithelial integrity or increase HSV-2 susceptibility. A) HSV-2 enhancement model. Balb/C mice (n = 40 per group) were depo-treated 7 d before starting vaginal application of each formulation daily for 7 d, followed by HSV-2 G challenge 12 h post-last gel. Percent of uninfected animals over time is shown (*P<0.05 vs. D-PBS, Fisher's exact test). B) Epithelial integrity testing. Depo-treated (vaginal dosing) and fasted (rectal dosing) mice were treated with MZC, D-PBS, or Gynol II. At 1, 6 and 24 h post-application, mice were euthanized, and the entire reproductive or rectal tract was surgically excised for morphological analysis. Pictures (20× magnification) are representative of six sections from two to three animals per group.

*In vivo* safety was evaluated in an HSV-2 infection enhancement model. Following repeated vaginal gel application, mice were challenged with a suboptimal inoculum of 2×10^3^ pfu/mouse that infects only 50% of the control animals (D-PBS-treated) [14]. Gynol II, but not MZC, enhanced the susceptibility of mice to HSV-2 infection (p<0.0001 vs D-PBS; Fig. 2A). In contrast, MZC treatment significantly decreased infection in this model (p = 0.0006 vs D-PBS).

Histological examination of cervicovaginal and rectal mucosae after single MZC application to mice revealed no signs of damage to the epithelial architecture (Fig. 2B). As expected, Gynol II profoundly damaged the cervicovaginal (peak damage at 6 h, repaired by 24 h) and rectal (peak damage at 1 h, repaired by 6–24 h) tissues as evidenced by epithelial sloughing and exposure of the lamina propria [23], [34].

### MZC exhibits potent and broad *in vitro* antiviral activity against HIV and prevents vaginal SHIV-RT infection in macaques

MZC showed broad antiviral activity against HIV independent of clade, tropism or phenotype (Table 2 and Table S1). To evaluate activity without the confounding influence of the moderate *in vitro* antiviral effect of CG [28], we replaced the supernatant containing MZC or CG with fresh stimulation media 18 h after adding virus. Under these conditions, CG exhibited no antiviral activity while MZC was still potent (IC_50_ values average ∼1 nM based on MIV-150 concentration or ∼ 0.0003 (1/30000) based on gel dilution since it is being compared to CG). These potent IC_50_ values combined with the lack of toxicity (CC_50_>0.05, the lowest dilution tested) result in high therapeutic index (TI) values (Table 2) against a spectrum of HIV strains including multidrug resistant strains/clones containing multiple DRMs. The only exceptions were two isolates (S18-7d7 and OL-1/4(II)d4) that each contained a combination of two NNRTI mutations that decreased MIV-150 susceptibility (K101E+Y181I and K103N+L100I, respectively) (Table 2 and Table S1).

**Table 2:** Antiviral Activity of MZC and CG against HIV-1 in PBMCs.

HIV-1	IC_50_ gel dilution (95% confidence interval)		TI (CC_50_>0.05)	
	MZC	CG	MZC	CG
NL4-3	0.0002 (0.00008 to 0.0007)	∼0.001	>250	>50
92UG029	0.0004 (0.0003 to 0.0005)	>0.0025	>125	ND
91US056	0.0004 (0.0002 to 0.0007)	>0.0025	>125	ND
92BR014	0.0003 (0.0001 to 0.0007)	>0.0025	>166.7	ND
92HT593	0.0001 (0.00005 to 0.0002)	>0.0025	>500	ND
97ZA009	0.0001 (0.00009 to 0.0002)	>0.0025	>500	ND
97USNG30	0.0006 (0.0005 to 0.0009)	>0.0025	>83.3	ND
96USNG31	0.0006 (0.0002 to 0.003)	>0.0025	>83.3	ND
CMU06	0.0001 (0.00005 to 0.0002)	>0.0025	>500	ND
92TH020	0.00006 (0.00004 to 0.0001)	>0.0025	>833.3	ND
93TH051	0.0004 (0.0002 to 0.001)	>0.0025	>125	ND
35764-2	0.00008 (0.00003 to 0.0002)	0.0004 (0.0002 to 0.0011)	>625	>125
7295-1	0.0005 (0.0004 to 0.0007)	0.001 (0.0002 to 0.009)	>100	>50
29129-2	0.0002 (0.00009 to 0.0003)	>0.0025	>250	ND
56252-1	0.0005 (0.0002 to 0.0014)	>0.0025	>100	ND
4755-5	0.0002 (0.0001 to 0.0005)	>0.0025	>250	ND
1617-1	0.0001 (0.00006 to 0.0004)	0.0005 (0.0002 to 0.0017)	>500	>100
7324-4	0.0003 (0.0002 to 0.0005)	>0.0025	>166.7	ND
7324-1	0.0001 (0.00007 to 0.0003)	>0.0025	>500	ND
8415-2	0.0004 (0.0001 to 0.0012)	>0.0025	>125	ND
6463-13	0.00004 (0.00002 to 0.0001)	>0.0025	>1250	ND
7136-1	0.0003 (0.0001 to 0.0009)	>0.0025	>166.7	ND
V16770-2	0.0005 (0.0004 to 0.0007)	0.0007 (0.0003 to 0.0018)	>100	>71
V17763-5	0.0004 (0.0001 to 0.0016)	0.0004 (0.0001 to 0.0007)	>125	>125
W1023892-2	0.0002 (0.00006 to 0.0005)	0.0005 (0.0002 to 0.0015)	>250	>100
J18-1(2)	0.0002 (0.0001 to 0.0007)	>0.0025	>250	ND
S18-7d7	>0.0025	>0.0025	ND	ND
OL-1/4(II)d4	>0.0025	>0.0025	ND	ND
C18-15d7	0.0015 (0.0011 to 0.0019)	>0.0025	>33.3	ND

ND =  Not determined

**Figure 3:**
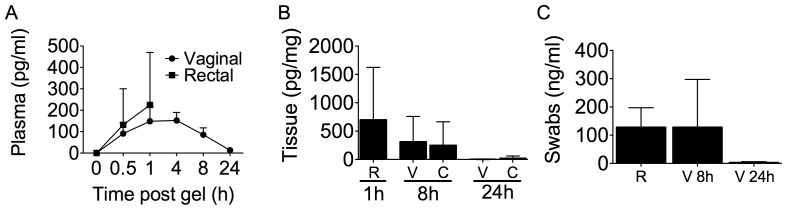
MIV-150 local and systemic levels after vaginal and rectal application. Macaques were treated vaginally with MZC daily for 2 wks or once rectally. A) MIV-150 was measured by LCMS/MS in the plasma of vaginally (n = 6, filled circles) and rectally (n = 6, filled squares) treated macaques at various time points after the last gel application. B) Tissue biopsies (n = 12 per time point/type of tissue) and C) swabs (n = 6 per condition) were collected at the indicated times following the last gel and MIV-150 was measured (by LCMS/MS for tissues and RIA for swabs). The mean (± SD) concentrations of MIV-150 for each treatment group are shown (rectal, R; vaginal, V; cervical, C).

In macaques treated vaginally or rectally with MZC, we quantified MIV-150 in plasma, mucosal tissues, and swabs. MIV-150 was detected in the plasma within 0.5 h of MZC treatment, regardless of the route of administration (Fig. 3A). There was no significant difference between plasma MIV-150 levels 0.5–1 h after vaginal and rectal application (of 2 and 3 ml gel, respectively). After vaginal application (where later time points were measured), plasma MIV-150 peaked between 1–4 h post-gel and then declined to undetectable levels within 24 h with a half-life of approximately 8 h. We have previously shown that MIV-150 has an IC_50_ and IC_90_ of 0.9 nM and 1.9 nM respectively against SHIV-RT in PBMCs [35]. MIV-150 could be detected in the cervical (60× IC_50_ and 30× IC_90_) and vaginal (6× IC_50_ and 3× IC_90_) tissues 8 h post vaginal MZC application (Fig. 3B), but this was significantly reduced within 24 h (vaginal p = 0.0030 and cervix p = 0.0237). MIV-150 was present in vaginal swabs at high concentrations (1000× IC_50_) 8 h post-gel but had declined below the limit of detection by 24 h (Fig. 3C). In rectally treated animals, MIV-150 was detected in both the rectal tissue and rectal fluid 1 h after gel administration (Fig. 3B and 3C).

**Figure 4:**
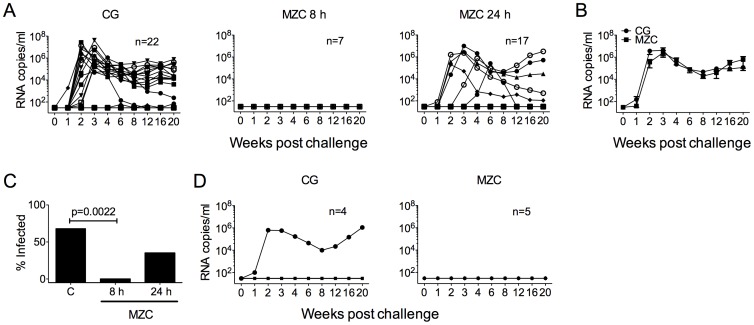
MZC completely protects against SHIV-RT infection vaginally for up to 8 h and rectally for 1 h. A) MZC or CG was administrated vaginally daily for 2 wks followed by vaginal challenge 8 or 24 h after the last gel application. The number of animals in each treatment group is indicated. For CG, this includes 8 real time and 14 historical controls. Plasma viral loads for each animal are shown over time. B) Mean (± SEM) plasma viral load of infected animals from each group in (A). C) The percent infection in each of the different treatment groups. D) MZC or CG was administrated once rectally followed by rectal challenge 1 h later. Plasma viral loads are shown for each animal over time. The number of animals in each treatment group is indicated.

MZC reduced SHIV-RT infection by the vaginal route (Fig. 4 and Table S2) with 6/8 real time CG controls becoming infected (75%, Table 3). This frequency of infection is slightly higher but not significantly different from the overall infection rate (64%) in CG treated animals from our previous studies of prototype and modified gels [12], [13], [35]. MZC provided complete protection (0/7 infected, 100% protection) when applied 8 h before challenge (Fig. 4A), but 47% when the animals were challenged 24 h after application (6/17 infected). Animals that became infected in the 24 h group exhibited similar viral loads overall to the infected CG controls (Fig. 4B). Protection in the 8 h group was highly significant vs CG (p = 0.0022 Fig. 4C), and the 47% protection after 24 h approached but did not reach significance (p = 0.0566) (Table 3). DRMs were not detected in the RT gene of SHIV-RT circulating in infected animals (Table S3).

**Table 3:** Summary of the efficacy data in the SHIV-RT macaque model testing prototype and modified gels.

Gel	Version	Number of Applications	Gel Dosing Relative to Challenge	Protection vs. CG (%)		Infected/Challenged		P value vs. CG
Vaginal Challenge								
MZC*	prototype	14 daily	8 h	100	85.2	0/7	2/21	0.0019
MZC*	prototype	14 daily or EOD	24 h	77.8		2/14		
CG*	prototype	14 daily	8–24 h	N/A		9/14		N/A

MZC	modified	14 daily	8 h	100		0/7		0.0022
MZC	modified	14 daily	24 h	47		6/17		0.0566
						
CG	modified	14 daily	8–24 h	N/A		6/8	15/22	N/A
	prototype	14 daily	8–24 h	N/A		9/14		N/A
Rectal Challenge								
MZC	modified	1	1 h	100		0/5		0.45
CG	modified	1	1 h	N/A		1/4		N/A
CG**	prototype	1	8–24 h	83.3		5/6		
MC***		1	0.5–4 h	100		4/4		

N/A =  Not Applicable

EOD =  Every other day

*Published data (reference 6), **Published data (reference 13), ***Published data (reference 29).

Prior studies using prototype MZC rectally revealed that there was less protection rectally than vaginally [13], possibly due to the more efficient transmission of immunodeficiency viruses across the rectal mucosa. Because rectal microbicide gels are likely to be used in a coitally dependent (on demand) manner in which they double as lubricants (most probably needing intrarectal application with rectal applicators), we decided to apply modified MZC only 1 h prior to challenge. Unfortunately, low-level infection in the CG-treated group (1/4 infected) resulted in an inability to detect protection by MZC gel (p = 0.45) even though none of the animals (0/5) became infected (Fig. 4D). Of note, prior studies showed that this SHIV-RT inoculum infects 100% of the animals when administered in the presence of an inert MC placebo gel and that there was limited impact of CG on infection when applied 8–24 h prior to rectal challenge (Table 3 [13], [23]). Thus, the blocking effect of CG against rectal SHIV-RT infection when given 1 h prior to challenge did not allow us to distinguish the activity of MZC. But MZC is safe rectally as it had no adverse (enhancing) effects that resulted in more animals becoming infected compared to the CG controls.

### MZC protects mice against vaginal and anorectal HSV-2 infection

**Figure 5:**
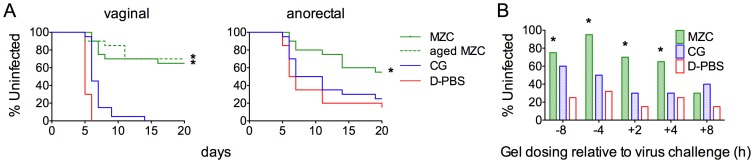
MZC protects mice against vaginal and anorectal HSV-2 infection. A) Depo-treated (vaginal) or untreated (anorectal) Balb/C mice were challenged with 10^6^ pfu HSV-2 10 min after applying the indicated formulations (n = 20/formulation). The percentages of uninfected animals over time, based on symptoms, are shown for each treatment group (*P<0.05 vs. D-PBS and CG, Fisher's exact test). B) Depo-treated Balb/C mice were treated with 10 µl of the indicated formulations intravaginally at different time points before or after HSV-2 challenge with 5×10^3^ pfu (n = 20/formulation). The percentages of uninfected animals are shown for each time of gel dosing relative to challenge (*P<0.05 vs. D-PBS, Fisher's exact test).

MZC applied 10 min before challenge, effectively blocked HSV-2 infection in stringent, high viral dose (10^6^ pfu) vaginal (1000 50% lethal dose (LD_50_)) and anorectal (10 LD_50_) mouse models. There was a significant decrease in infection after vaginal (65% uninfected, p <0.0001 vs. D-PBS or CG) and anorectal (55% uninfected, p = 0.0187 vs. D-PBS) challenge (Fig. 5A). Supporting initial stability testing (Table 1), MZC that was aged for 7 months at 40°C was comparably active against vaginal challenge (Fig. 5A). Under less stringent conditions (5×10^3^ pfu/mouse), we determined the window of protection by applying MZC, CG or D-PBS at different times before or after viral challenge. A significant decrease in the percentage of mouse infection vs. D-PBS was observed when MZC was administered vaginally between 8 h before (p = 0.0038) and 4 h after (p = 0.0248) virus challenge (Fig. 5B). A blocking effect of CG was seen with this lower inoculum. When the same experiment was performed for HSV-2 anorectal challenge, no significant protection was seen when MZC was applied at any of the time points tested (data not shown), except for 10 min before HSV-2 challenge (Fig. 5A).

### Breadth, high potency and durability of the anti-HPV effect of MZC in mice

**Figure 6:**
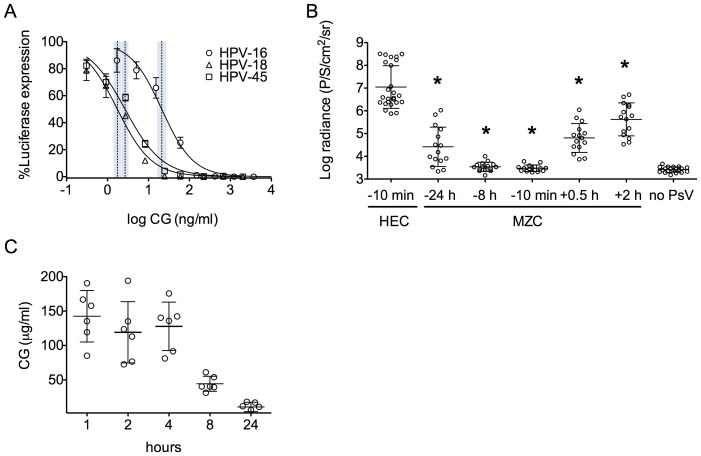
MZC has potent anti-HPV activity *in vitro* and prevents HPV-16 PsV vaginal infection in mice. A) The anti-HPV-16 (open circles), 18 (open trianbles) and 45 (open squares) IC_50_ values (shown as a vertical dotted line within the 95% confidence interval) were estimated using the luciferase assay in HeLa cells. All gel dilutions were tested in triplicate. B) Depo-treated Balb/C mice were given the indicated formulations (HEC placebo or MZC) intravaginally at different time points before HPV-16 PsV challenge (15 animals/treatment). *In vivo* luciferase expression was measured 24 h after challenge using the IVIS Lumina and is expressed as mean luminescence in photons per second per centimeter square per steridian ± SD (*P<0.05 vs. HEC, Mann Whitney U test). C) CG levels (mean μg/ml ± SD) in vaginal washes from mice treated intravaginally with MZC were measured by ELISA at 1, 2, 4, 8 and 24 h post-gel (n = 6 per time point).

CG has been shown to block HPV PsV infection [15], [17], [18]. *In vitro*, MZC was active against three of the most predominant high-risk HPV genotypes (16, 18 and 45) at high gel dilutions, with IC50 values at dilutions between 1/10^7^ and 1/10^8^ (corresponding to CG concentrations between 1 and 20 ng/ml) (Fig. 6A). None of the MZC dilutions tested in HeLa cells were toxic (CC_50_>0.017, based on gel dilution), which translates to TI values >17,000. Since MZC was more potent against HPV-18 and 45 than HPV-16, HPV-16 was used as a more stringent test for *in vivo* use.

MZC fully protected mice when applied 8 h before vaginal HPV-16 PsV challenge and still significantly reduced infection when applied 24 h before or 2 h after challenge (Fig. 6B; earliest/latest time points tested). This coincided with the levels of CG in vaginal washes becoming lower after 24 h (10.7±6.8 µg/ml), with an average of 44.5±10.9 µg/ml CG being present after 8 h (Fig. 6C).

**Figure 7:**
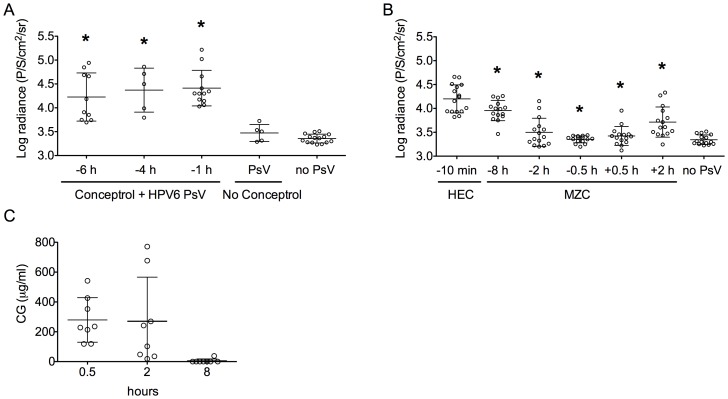
MZC prevents HPV-16 PsV anorectal infection in mice. A) Ketamine/xylazine-anesthetized Balb/C mice were treated in the anorectal area with Conceptrol or D-PBS (no Conceptrol). Mice were challenged with HPV-16 PsV to determine the optimal time for conceptrol treatment that gives the best luminescence signal. B) Ketamine/xylazine-anesthetized Balb/C mice received Conceptrol in the anorectal area and were treated with the indicated formulations (HEC placebo for HPV-16 PsV control or MZC) in the anorectal area at different time points before and after HPV-16 PsV challenge (15 animals/treatment). (A and B) *In vivo* luciferase expression was measured 48 h after challenge using the IVIS Lumina and is expressed as mean luminescence in photons per second per centimeter square per steridian + SD (*P<0.05 vs. HEC, Mann Whitney U test). C) CG PK in rectal washes from mice treated with MZC formulation was determined at 0.5, 2, and 8 h (n = 6 or 8 per time point) after MZC application. The graph shows CG concentration (mean μg/ml ± SD) per time point.

To evaluate the activity of MZC against anorectal HPV infection, we established an anorectal HPV16 PsV murine model based on the vaginal model [17] and our experience with the anorectal HSV-2 mouse model [14]. As anticipated from the rapid damage observed after rectal application of Gynol II (Fig. 2B), we achieved consistent HPV-16 PsV infection when Conceptrol (4% Nonoxynol 9 like Gynol II) was applied rectally 1–6 h before challenge (Fig. 7A). Detection of the PsV by luminescence (radiance) was significantly higher (p<0.0001) under this condition (or even when applying Conceptrol 6 or 4 h before PsV challenge) compared to animals not treated with Conceptrol (Fig. 7A). Although there was no significant difference in radiance between 6, 4 and 1 h Conceptrol pre-treatment (p> 0.306), we decided to use the 1 h timing since the radiance values were more consistent at this time. Under these conditions, MZC protected against HPV-16 PsV anorectal challenge when applied between 2 h before and 2 h after HPV-16 PsV challenge (p<0.0006) (Fig. 7B). This corresponds to CG levels in rectal swabs that averaged 271 µg/ml (Fig. 7C). Notably, although CG was not detectable in the rectal swabs after 8 h (probably due to the dilution factor and the ELISA LLOQ), animals were still significantly protected against infection (albeit to a lesser degree than at the earlier time points).

## Discussion

Infection with either of the highly prevalent STIs HSV-2 or HPV enhances susceptibility to HIV acquisition [1], [2]. MPTs that target multiple organisms offer a unique, single product platform to prevent these intersecting diseases. We have modified a combination MZC gel for use in humans that is safe and exhibits broad spectrum, potent and durable protection against HIV, HSV-2, and HPV. Moreover, MZC was modified with a co-solvent more appropriate for use in humans (propylene glycol vs. DMSO used in the prototype) in preparation for clinical testing.

MZC was safe in all the *in vitro* and *in vivo* assays performed. The (a) unaltered TEER values, (b) lack of toxicity to *Lactobacillus sp.* and *C. albicans*, (c) absence of damage to the architecture of mucosal tissues and (d) lack of increase in susceptibility to HSV-2 correlates with results obtained with the modified ZC gel [23]. Further supporting the safety of this gel for both vaginal and rectal application are the data showing protection against SHIV-RT following daily vaginal application and the lack of increased rectal SHIV-RT infection with gel use. MZC contains only 0.002% MIV-150 with 0.3% ZA and 3% CG. ZA is generally recognized as safe (GRAS), and no toxicity has been seen in rabbits after daily vaginal dosing with a 90 mM zinc solution or zinc-loaded sponges for 10 d [36], [37]. Some adverse effects have been documented after oral, nasal or vaginal administration of zinc salts [38], [39], [40]. However, the events were linked to doses of zinc salts (200 mM) that far exceed those in our MZC formulation (14 mM). CG is also considered GRAS, has been shown to be safe and acceptable for topical use in humans [41], [42], [43], [44], [45], [46], [47], has good physical/rheological properties for a microbicide [14] and possesses intrinsic antiviral activity [15], [16], [17], [18].

Vaginal susceptibility to SHIV-RT was not significantly affected by the altered gel composition. Infection frequency in the CG-treated group was not significantly higher (p = 1.000) with modified (6/8, 75%) compared to prototype (9/14, 64.3%) gel. Modified MZC gel exerted time dependent protection against SHIV-RT. In accordance with this, significantly more MIV-150 was detected in the vaginal (p = 0.003) and cervical (p = 0.0237) tissues, the vaginal swabs (p = 0.0115) and the plasma (p = 0.0139) at 8 h compared to 24 h post-last gel. No infections occurred when animals were challenged vaginally 8 h after the last MZC application for both the prototype and modified formulations (0/7 vs. 0/7) [12], [13]. Although there was no significant difference at 24 h (2/14 vs. 6/17, p = 0.239), the modified formulation was not as protective as the prototype at this time point. In agreement with the infection data at 8 h, the two formulations resulted in similar levels of MIV-150 in the vaginal tissue 8 h post-last gel (mean ± SD 0.3347±0.1872 for prototype vs. 0.2958±0.1112 for modified, p = 0.6451) as well as in the vaginal swabs (mean ± SD 74.6±69.8 vs. 57.3±75.4, p = 0.7922). Notably, significantly less MIV-150 was detected in the cervix 8 h after application of the modified compared to prototype gel despite retaining complete protection (mean ± SD 0.6274±0.2439 vs. 0.03505±0.007339, p = 0.0043). Although no significant differences in tissue pharmacokinetic (PK) were found 24 h post-last gel, less MIV-150 was detected in the vagina 24 h after application of modified MZC vs. prototype, and significantly less MIV-150 was present in the vaginal swabs (mean ±SD 2.8±5.7 vs. 22.3±21.3, p = 0.0181) explaining the finding of reduced protection by modified MZC gel at the 24 h time point and the absence of gel in the vagina by MRI. Importantly, a single application of the prototype MZC gel fully protects when administered 8 h before vaginal SHIV-RT challenge in macaques [13]. The rationale for using the repeated dosing regimen in this study was to simultaneously test safety alongside the gel's efficacy in macaques. Daily application allowed us to observe any detrimental effects that could cause an enhancement in SHIV-RT infection as is seen in the increased susceptibility model for HSV-2.

Unfortunately, we were not able to observe a significant effect of MZC gel on rectal SHIV-RT infection due to the blocking activity of CG when applied close to the time of challenge. The activity of CG in this model is likely a combination of a non-specific barrier effect of the gel due to coating of the epithelium coupled with some characteristic polyanion blockade of cell-virus interactions [48]. Its time dependence is clear: 1/4 animals became infected when the gel was applied 1 h before challenge compared to 2/3 with gel applied 8 h pre and 3/3 infected with gel applied 24 h pre challenge [13]. The anti-HIV effect of CG in this model is further supported by earlier work demonstrating reliable infection of placebo MC gel-treated animals (4/4) [30]. As a result of the CG blocking effect, nothing can be formally concluded from these data about the gel's efficacy rectally, but MZC did not enhance infection when used close to the time of challenge indicating its safety as a rectal product.

The anti-HIV activity of MZC covers a broad array of primary isolates *in vitro*, including those from different clades with distinct phenotypes based on co-receptor usage and cell tropism. The only viruses tested in this study which were not susceptible to inhibition by MZC possess double NNRTI-associated mutations: OL-1/4(II)d4, a mutant with more than 20 DRMs, expresses an RT with K101E and Y181I, and S18-7d7 expresses an RT with L100I and K103N. However, three other isolates with double or triple NNRTI-associated RT mutations (V16770-2, V17763-5, W1023892-2) were efficiently blocked. Importantly, these *in vitro* experiments using cell lines may exclude any direct or indirect immunomodulatory effects of ZA [49], [50], [51], [52], [53], which could boost the gel's potency against isolates with DRMs. Additionally, the full consequences of HIV drug resistance in the context of prevention are not yet fully understood and could be less significant than in a treatment setting [54], [55].

We have previously shown that combining ZA with CG results in synergistic antiviral activity against HSV-2 [14]. In this first testing of MZC against HSV-2 in the mouse model, we demonstrated significant anti-HSV-2 activity vaginally under stringent conditions in which mice were inoculated with 10^6^ pfu of virus. Other microbicide candidates with anti-HSV-2 activity have not shown this level of protection in mice under such stringent conditions [56], [57], [58]. Protection against HSV-2 by MZC lasted up to 8 h after gel application when challenging with 5×10^3^ pfu. Post-challenge protection in this model lasted up to 4 h. The *in vivo* activity of MZC against HSV-2 in this study correlates well with the activity of other zinc-containing formulations we have studied [14], [23]. Not surprisingly, due to the lack of anti-HSV-2 activity by the NNRTI MIV-150, the frequency of infection seen with MZC (7/20 vaginal and 9/20 anorectal) is not significantly different (p>0.320) from that seen for prototype (5-6/18 vaginal and 5-8/20 anorectal) [14] or modified (4/20 vaginal and 8/20 anorectal) ZC gels [23].

HPV-16 and 18 are the most oncogenic HPV genotypes, associated with 75% of cervical cancers, 50% of vulvar cancer, 50–75% of vaginal cancers and 80% of anal cancer [3]. Modified MZC was potent *in vitro* against HPV-16 and 18 as well as 45, demonstrating a good breadth of protection. The IC_50_ values against HPV are about a thousand-fold more potent than those described for CG against HIV [28]. The CG vaginal PK data indicate that CG concentrations in the vaginal lumen above 50 µg/ml (observed 8 h after gel application) may be needed to achieve full protection *in vivo* as shown in the mouse model at the same time point. However, the lower concentrations (∼10 µg/ml) seen at 24 h after gel application, may still significantly decrease infection, representing more than a thousand-fold higher CG concentration compared to the *in vitro* IC_50_ value against HPV-16 PsV. The *in vivo* model shows about a 50% reduction in infection when concentrations of CG are about a 1000-fold higher than the *in vitro* IC_50_ values. This difference could be due to different multiplicities of infections between the *in vivo* and *in vitro* model what can impact the antiviral activity of CG. It has been shown that the IC_50s_ for the various types of CG occur under conditions where there is a slight mass (or molar) excess of CG over L1 (HPV capsid protein) [15]. If more virus is added, the IC_50_ is expected to increase. A partial but significant reduction in infection was also observed when the gel was applied 2 h after PsV challenge. These results are in accordance with other *in vitro* data indicating that CG may block HPV post-challenge [15]. Another important result that supports the testing of CG as a potential microbicide against HPV comes from the Carraguard Phase 3 trial. The prevalence of high-risk HPV infection observed in compliant users was significantly lower compared to compliant placebo users [16].

To our knowledge, this is the first description of an anorectal mouse model for infection with HPV PsV. HPV infection in the anorectal area has been widely documented in humans [59]. Our findings support a model in which papillomaviruses from different species and with different tissue tropisms may share the same initial interactions with basal epithelial cells [60], allowing PsV binding and reporter genome delivery into these cells regardless of their anatomical location (vaginal, anorectal or dermal).

The efficacy results in the different animal models tested herein demonstrate the high potency and durability of the antiviral effect of MZC against the three most important viral STIs. The results suggest the potential for MZC to be not only a clinically useful, coitally dependent (on demand) vaginal microbicide with a broad window of protection (at least for vaginal application), but also an effective method of post-exposure prophylaxis for the prevention of HPV and HSV-2. Rectally, MZC needed to be applied much closer to the time of challenge to be effective against each virus. However, rectal microbicide gels will likely double as lubricants (which may also increase their acceptability [61]), and application close to the time of exposure is expected.

The battery of assays, used in this manuscript to evaluate the microbicide candidate, has been widely described in the literature [12], [13], [14], [17], [23], [24], [28], [29], [30], [34], [35], [57], [62], [63], [64], [65], [66], [67], [68]. Additional work has been done in terms of preclinical evaluation of MZC safety including a 14 day rabbit vaginal irritation assessment without any adverse effects (unpublished). We recognize the limitations of the assays described in this manuscript, including the need for validation in human trials [65], [66], [69]. To this end a Phase 1 safety trial is planned to start early 2014 to evaluate safety and PK of MZC. The *in vivo* models described in this paper, using depo treatment, extremely high viral doses for challenge and treatment with surfactant agents to expose viral targets, represent highly stringent models. All these conditions may favor the failure of a promising microbicide agent, but the MZC formulation still showed a potent antiviral activity.

While this is a proof of concept study, there are some caveats related to gel formulations and poor adherence that has been seen in some vaginal microbicide clinical trials [45], [70]. Additionally, gels are designed for use around time of intercourse. We are currently exploring the formulation of MZC in intravaginal rings and nanofibers to provide sustained release of the APIs and potentially improve their acceptability. Taken together, our results demonstrate that MZC is a promising microbicide combination that should be advanced for clinical testing.

## Supporting Information

Figure S1 **MRI detection of MZC spreading in the macaque reproductive tract.** MRI was performed 2 and 24 h after vaginal MZC administration in two macaques. Transverse (upper panels) and sagittal (lower panels) images were taken to assess spread of the gel throughout the vaginal vault. The arrows show the detection of MZC gel at 2 h but not at 24 h. The images shown in the panel are representative of 20 tranverse and 20 sagittal images.
(DOCX)

Table S1 **Summary of HIV-1 isolates and clones used to test the** ***in vitro*** **anti-HIV activity of MZC.**
(DOCX)

Table S2 **Summary of rhesus macaques.**
(DOCX)

Table S3 **Modified MZC applied vaginally does not select for NNRTI-resistant variants.**
(DOC)
