# Supplementary material for: A Potent Combination Microbicide that Targets SHIV-RT, HSV-2 and HPV
Source: PLoS One. 2014 Apr 16;9(4):e94547. doi: 10.1371/journal.pone.0094547 (PMC3989196; doi:10.1371/journal.pone.0094547)
Supplement: Figure S1 — MRI detection of MZC spreading in the macaque reproductive tract. MRI was performed 2 and 24 h after vaginal MZC administration in two macaques. Transverse (upper panels) and sagittal (lower panels) images were taken to assess spread of the gel throughout the vaginal vault. The arrows show the detection of MZC gel at 2 h but not at 24 h. The images shown in the panel are representative of 20 tranverse and 20 sagittal images. (DOCX) [file pone.0094547.s001.docx]

**
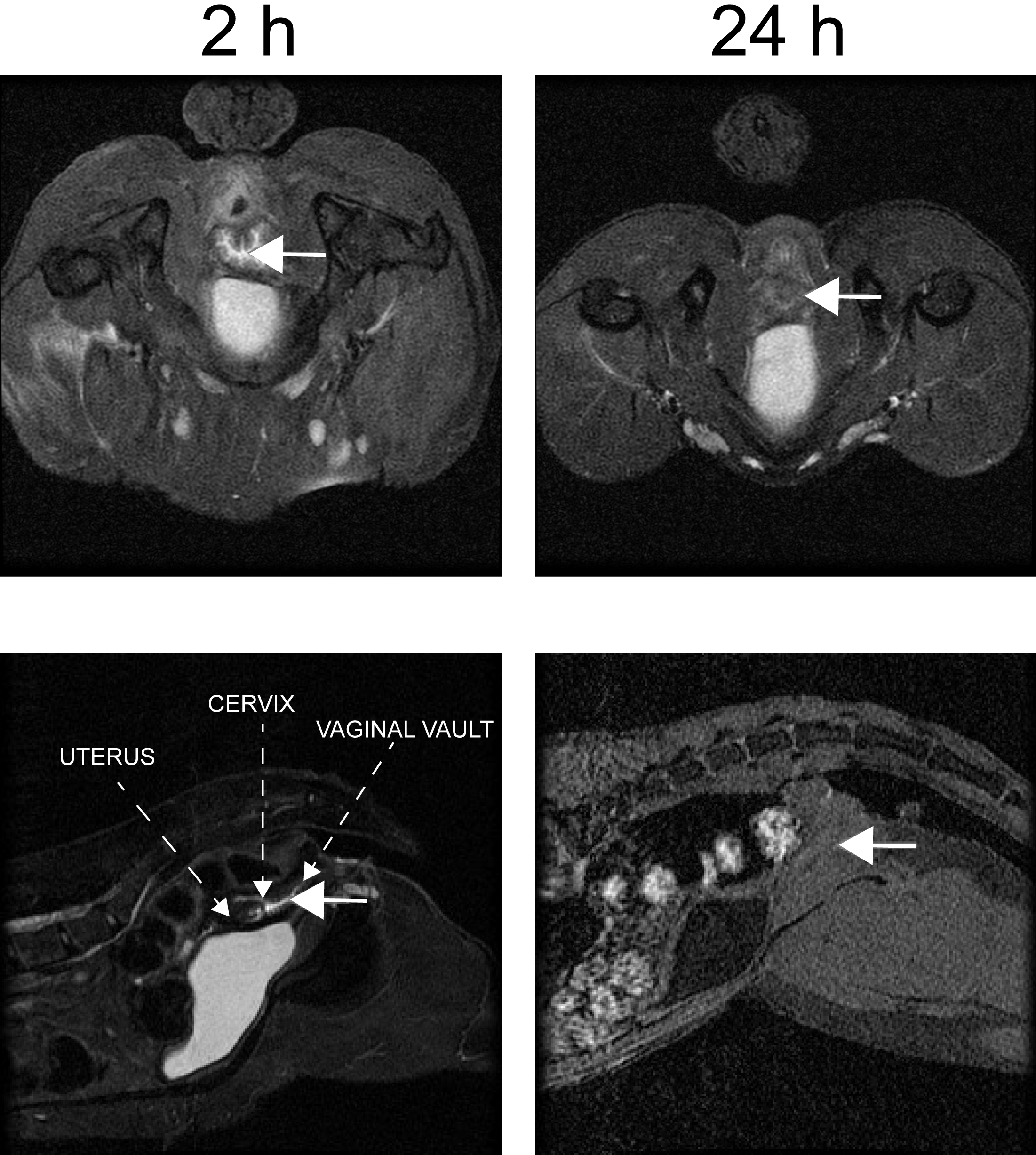
**

**Figure S1. MRI detection of MZC spreading in the macaque reproductive tract.** MRI was performed 2 and 24 h after vaginal MZC administration in two macaques. Transverse (upper panels) and sagittal (lower panels) images were taken to assess spread of the gel throughout the vaginal vault. The arrows show the detection of MZC gel at 2 h but not at 24 h. The images shown in the panel are representative of 20 tranverse and 20 sagittal images.
