# Supplementary material for: A Potent Combination Microbicide that Targets SHIV-RT, HSV-2 and HPV
Source: PLoS One. 2014 Apr 16;9(4):e94547. doi: 10.1371/journal.pone.0094547 (PMC3989196; doi:10.1371/journal.pone.0094547)
Supplement: Table S1 — Summary of HIV-1 isolates and clones used to test the in vitro anti-HIV activity of MZC. (DOCX) [file pone.0094547.s002.docx]

**Table S1. Summary of HIV-1 isolates and clones used to test the *in vitro* anti-HIV activity of MZC.**

| **HIV-1 or SHIV strain/ clone** | **Clade** | **Coreceptor** | **RT mutations** | |
| --- | --- | --- | --- | --- |
|  |  |  | **NRTI** | **NNRTI** |
| NL4-3* | B | X4 | - | - |
| 92UG029* | GAG/ENV-A | X4 | - | - |
| 91US056* | ENV-B | R5 | - | - |
| 92BR014* | GAG/ENV-B | R5/X4 | - | - |
| 92HT593* | ENV-B | R5/X4 | - | - |
| 97ZA009* | GAG/ENV-C | R5 | - | - |
| 97USNG30* | ENV-C | R5 | - | - |
| 96USNG31* | ENV-C | R5/X4/R3 | - | - |
| CMU06* | ENV-E |  | - | - |
| 92TH020* | ENV-E | R5 | - | - |
| 93TH051* | ENV-E | R5/X4 | - | - |
| 35764-2^†^ | RT-B |  | 75I, 77L, 116Y, 151M | - |
| 7295-1^†^ | RT-B |  | 67N, 70R, 215F, 219Q, 184V, 69N, 218E | - |
| 29129-2^†^ | RT-B |  | 41L, 67G, 210W, 215Y, 184V | 103N |
| 56252-1^†^ | RT-B |  | 70R, 77L, 116Y, 151M, 65R, 75I, 115F, 39A, 44D | 103N |
| 4755-5^†^ | RT-B |  | 41L, 67G, 210W, 215Y, 184V, 69D, 39A, 43A, 44D, 118I | - |
| 1617-1^†^ | RT-B |  | 70G, 184V, 69K, 62V, 77L, 116Y, 115M, 75I, 115F | - |
| 7324-4^†^ | RT-B |  | 67N, 70R, 215F, 219E, 39A, 203D | - |
| 7136-1^†^ | RT-B |  | 65R | - |
| 8415-2^†^ | RT-B |  | 184V, 65R | - |
| 7324-1^†^ | RT-B |  | 41L, 67N, 70R, 215F, 219E, 69N, 39A, 203D | - |
| V16770-2^‡^ | RT-B |  | 210W, 215Y | 227L, 230L |
| V17763-5^‡^ | RT-B |  | 41L, 215Y | 101P, 103N |
| W1023892-2^‡^ | RT-B |  | 67N, 69N, 215S, 219Q | 101E, 138K, 181C |
| J18-1 (2)8/30/04^§^ | B |  | 41L, 44D, 69D, 118I, 210W, 215D | 108I |
| S18-7d7^§^ | B |  | 70R | 100I, 103N |
| OL-1/4(II)d4^§^ | B |  | 41L, 67D/N, 118I, 184V/I, 210 L/G/M/R/V/W, 215C/Y, 219E | 101E, 181I |
| C18-15d7^§^ | B |  | 67N, 69D, 215S/F, 219Q | 101Q, 188L |
| SHIV-RT^¶^ | RT-B | R5 | - | - |

***** obtained through the NIH AIDS Reagent Program, Division of AIDS, NIAID, NIH.

^†^ obtained through the NIH AIDS Reagent Program, Division of AIDS, NIAID, NIH from Dr. Robert Shafer.

^‡^ provided by Dr. Shafer at Stanford University

^§^ provided by Dr. Markowitz at ADARC

^¶^ provided by Disa Böttiger, Medivir AB, Sweden
