# Supplementary material for: A Potent Combination Microbicide that Targets SHIV-RT, HSV-2 and HPV
Source: PLoS One. 2014 Apr 16;9(4):e94547. doi: 10.1371/journal.pone.0094547 (PMC3989196; doi:10.1371/journal.pone.0094547)
Supplement: Table S2 — Summary of rhesus macaques. (DOCX) [file pone.0094547.s003.docx]

**Table S2. Summary of rhesus macaques.**

| Animals ID | Gel | Time | Challenge Route | SIV DNA in PBMC^1^ | Plasma Viral Load | SIV Ab Response^2^ |
| --- | --- | --- | --- | --- | --- | --- |
| CA98 | MZC | 24h | Vaginal | + | + | + |
| GC12 | MZC | 24h | Vaginal | - | - | - |
| CM11 | MZC | 24h | Vaginal | - | - | - |
| CM08 | MZC | 24h | Vaginal | - | - | - |
| CM72 | MZC | 24h | Vaginal | - | - | - |
| CP34 | MZC | 24h | Vaginal | + | + | - |
| GD92 | MZC | 24h | Vaginal | - | - | - |
| DG89 | MZC | 24h | Vaginal | - | + | + |
| GA88 | MZC | 24h | Vaginal | - | - | - |
| CI94 | MZC | 24h | Vaginal | - | - | - |
| EF65 | MZC | 24h | Vaginal | - | - | - |
| IR47 | MZC | 24h | Vaginal | + | + | + |
| IR43 | MZC | 24h | Vaginal | + | + | + |
| IR50 | MZC | 24h | Vaginal | - | - | - |
| IR22 | MZC | 24h | Vaginal | - | - | - |
| EK86 | MZC | 24h | Vaginal | - | - | - |
| GV93 | MZC | 24h | Vaginal | + | + | + |
| HL67 | MZC | 8h | Vaginal | - | - | - |
| DD56 | MZC | 8h | Vaginal | - | - | - |
| FT48 | MZC | 8h | Vaginal | - | - | - |
| EE72 | MZC | 8h | Vaginal | - | - | - |
| FM70 | MZC | 8h | Vaginal | - | - | - |
| IR25 | MZC | 8h | Vaginal | - | - | - |
| IR33 | MZC | 8h | Vaginal | - | - | - |
| FI14 | CG | 8h | Vaginal | + | + | + |
| GJ79 | CG | 8h | Vaginal | - | - | - |
| GE23 | CG | 24h | Vaginal | + | + | + |
| DD29 | CG | 24h | Vaginal | + | + | + |
| GM30 | CG | 24h | Vaginal | - | - | - |
| GP49 | CG | 24h | Vaginal | + | + | + |
| EF46 | CG | 24h | Vaginal | + | + | + |
| IR45 | CG | 24h | Vaginal | - | - | - |
|  |  |  |  |  |  |  |
| HL60 | MZC | 1h | Rectal | - | - | - |
| GT59 | MZC | 1h | Rectal | - | - | - |
| HL53 | MZC | 1h | Rectal | - | - | - |
| IE84 | MZC | 1h | Rectal | - | - | - |
| IE79 | MZC | 1h | Rectal | - | - | - |
| IE89 | CG | 1h | Rectal | + | + | + |
| IE81 | CG | 1h | Rectal | - | - | - |
| HL56 | CG | 1h | Rectal | - | - | - |
| IC84 | CG | 1h | Rectal | - | - | - |

^1^DNA was tested at weeks 2-8.

^2^ELISA for Abs to SIV was performed at weeks 4-8 and compared with baseline.
