# Supplementary material for: A Potent Combination Microbicide that Targets SHIV-RT, HSV-2 and HPV
Source: PLoS One. 2014 Apr 16;9(4):e94547. doi: 10.1371/journal.pone.0094547 (PMC3989196; doi:10.1371/journal.pone.0094547)
Supplement: Table S3 — Modified MZC applied vaginally does not select for NNRTI-resistant variants. (DOC) [file pone.0094547.s004.doc]

**Table S3. Modified MZC** applied vaginally does not select for NNRTI-resistant variants.

| **Animal ID** | **L100I, K101P, K103N, V108I, I178L, V179I, Y181C, Y188L, G190E, P225H** |
| --- | --- |
| CA98 | 0 (16) |
| CP34 | 0 (16) |
| DG89 | 0 (19) |
| GV93 | 0 (17) |
| IR43 | 0 (17) |
| IR47 | 0 (16) |

The table lists the number of clones in which amino acid mutations conferring NNRTI resistance were detected. Parentheses indicate the total number of clones sequenced per animal.
